# High Resolution Scanning Electron Microscopy of Cells Using Dielectrophoresis

**DOI:** 10.1371/journal.pone.0104109

**Published:** 2014-08-04

**Authors:** Shi-Yang Tang, Wei Zhang, Rebecca Soffe, Sofia Nahavandi, Ravi Shukla, Khashayar Khoshmanesh

**Affiliations:** 1 School of Electrical and Computer Engineering, RMIT University, Victoria, Australia; 2 Faculty of Medicine, Dentistry, and Health Sciences, The University of Melbourne, Victoria, Australia; 3 School of Applied Sciences, RMIT University, Victoria, Australia; Queen’s University at Kingston, Canada

## Abstract

Ultrastructural analysis of cells can reveal valuable information about their morphological, physiological, and biochemical characteristics. Scanning electron microscopy (SEM) has been widely used to provide high-resolution images from the surface of biological samples. However, samples need to be dehydrated and coated with conductive materials for SEM imaging. Besides, immobilizing non-adherent cells during processing and analysis is challenging and requires complex fixation protocols. In this work, we developed a novel dielectrophoresis based microfluidic platform for interfacing non-adherent cells with high-resolution SEM at low vacuum mode. The system enables rapid immobilization and dehydration of samples without deposition of chemical residues over the cell surface. Moreover, it enables the on-chip chemical stimulation and fixation of immobilized cells with minimum dislodgement. These advantages were demonstrated for comparing the morphological changes of non-budding and budding yeast cells following Lyticase treatment.

## Introduction

The morphology of cells can reveal essential information about their type, structure, and condition. For example, apoptosis and necrosis are associated with cell surface alterations including shrinking, swelling, scaring, smoothing, loss of microvillus structures, and blebbing, etc [1]. Moreover, the surface of a cell can change in response to different chemical stimuli. For example, exposure to toxins such as hydrogen peroxide (H_2_O_2_) and alcohols can cause morphological changes to the cell surface [2]–[4]. Similarly, substances secreted from a cell may also lead to morphological changes in the adjacent cells. This is observed when chemotactic molecules such as chemokines induce rearrangements of cytoskeletal contractile elements in leukocytes, resulting in the extension of pseudopods enabling cell movement, or yeast mating initiated by pheromones which stimulate the growth of projections toward each other [5]–[8]. Besides, physical stimuli such as shear stress [9], electric or magnetic fields [10], [11] and variation of temperature [12] may also regulate the cell response and hence cause cell morphological changes.

Remarkably, ultrastructural analysis of cells provides more detailed information about their structure. Indeed, in clinical medicine it has been valuable in the differential diagnosis of tumors [13]–[15]. Pharmacological endeavors of drug discovery and investigating drug effects have also utilized ultrastructural cell analysis [16]–[18]. Furthermore, in fundamental biology, characterization of important biological structures such as presynaptic terminals, and examination of embryonic cell lineage differentiation has also been enabled [19], [20].

Environmental scanning electron microscopy (ESEM) has been widely used for studying the ultrastructure of biological samples [21]. ESEM works in a hydrated atmosphere and thus facilitates imaging of biological samples without prior preparation such as dehydration, critical point drying and conductive coating [21], [22]. However, the main disadvantage of ESEM is its low resolution compared to the conventional SEM [22]. Recently, ultra-high resolution, low vacuum SEM has been designed specifically to image charging or contaminating samples. Helix gaseous secondary electron detector has been incorporated to achieve unprecedented resolution in low vacuum modes [23], [24]. This enables SEM systems to achieve detailed information about the surface of biological samples with ultra-high resolution.

However, SEM imaging for many samples such as yeast and tumor cells represents a particular challenge. This is because these cells are non-adherent, and their immobilization requires complex fixation protocols that may lead to changes in the structure, morphology, and physical-chemical properties of the cells [25]–[27].

Microfluidic platforms enable the manipulation, sorting, and trapping of cells in microenvironments with resolutions that cannot be matched by existing techniques. Due to the laminar characteristics of the flow, microfluidic platforms facilitate the precise temporal and spatial control over the population of immobilized cells, concentration of perfused chemicals, and gradient of temperature within the medium [28]–[31]. Although enclosed microfluidic cell arrays for hydrodynamic trapping and dynamic analysis of cells have been reported [27], [32], [33], they are not suitable for interfacing with SEM. Dielectrophoresis exploits the motion of charged or neutral particles in non-uniform electric fields. It has been proven as a versatile tool for the rapid and efficient sorting, immobilization and characterization of cells for a variety of applications including diagnostics, drug discovery and investigating the functioning of cells under well-controlled conditions [34]–[40]. More importantly, dielectrophoresis can be readily used to interface cells with different analytical tools and techniques such as Raman spectroscopy and ESEM [41]–[43].

We have recently developed a protocol for interfacing non-adherent cells with ESEM [43]. The protocol involved three steps, including- (i) immobilizing cells between the microelectrodes under positive DEP force for 5 minutes, (ii) exposing cells to a weak electric field for 90 minutes to ensure their immobilization, and (iii) discharging the liquid from the micro-chamber using a pipette. However, this protocol had several issues including the long time required to prepare the sample, possible dislodgement of immobilized cells during the discharging step, and deposition of liquid residues (i.e. small molecules of glucose or sucrose) over the surface of cells during the discharging step. More importantly, it did not allow the on-chip stimulation, fixation and proper dehydration of immobilized cells, as the aspiration process could lead to significant dislodgement of cells. Additionally, implementing ESEM greatly compromised the resolution of the images, and hence made it difficult to obtain detailed information about the cell morphology and surface changes.

These limitations motivated us to develop a novel microfluidic based protocol for interfacing non-adherent cells with high-resolution SEM at low vacuum mode. The protocol enables rapid immobilization of the cells followed by drying of medium remained in the micro-chamber before SEM imaging. Desired media or chemicals can be applied to wash or stimulate the immobilized cells with minimum dislodgement. This not only accelerates preparation process but also avoids the deposition of chemical residues over the cell surface, which can compromise the imaging resolution. This technique enabled us to compare the morphological changes of non-budding and budding yeast cells following treatment with Lyticase.

## Materials and Methods

### Dielectrophoretic (DEP) System Design and Fabrication

**Figure 1:**
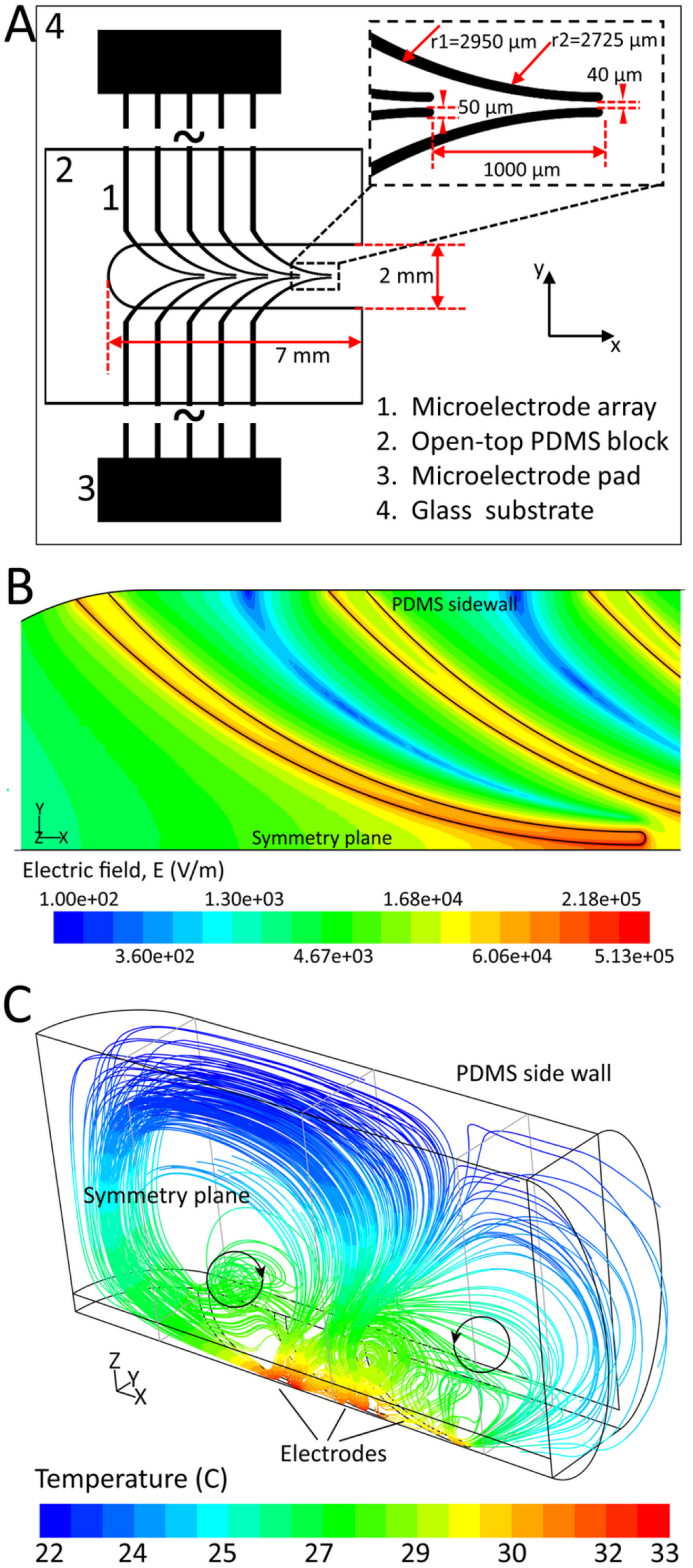
Specifications of the applied DEP systems: (A) an open-top PDMS block was assembled onto a DEP platform equipped with one microelectrode array, the inset shows the magnified image of one pair of the curved microelectrodes, the minimum gap of the electrode is 40 µm and the width of the electrod tip is 50 µm. (B) Contours of electric field at the levitation height of z = 10 µm. (C) The formation of vortices due to the electro-thermal effects, obtained under the medium conductivity of 0.03 S/m. The streamlines are colored according to the local temperature of the liquid. A maximum velocity of 54 µm/s was achieved along the tip of microelectrodes.

Figure 1A shows the plan view of the developed DEP system. A 5 mm thick polydimethylsiloxane (PDMS) block with a 7×2 mm notch was assembled onto the glass substrate, which accommodated the DEP microelectrode array. The microelectrodes were made by depositing thin layers of chromium/gold (100 nm/100 nm) using electron beam evaporation method and patterned using standard photolithography technique [44]. Curved microelectrodes were used as they produce strong non-uniform electric fields over the tip region and provide a large area for immobilization of cells [36], [45]. The microelectrodes have a tip width of 50 µm and a minimum gap of 40 µm at the tips while the spacing between the two consequential pairs is 1000 µm (Figure 1A inset).

### DEP System Analysis and Modelling

Assuming that yeast cells have a spherical structure, they experience a time-averaged DEP force as given below [46]:
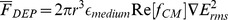
(1)where *r* is the radius of cells, *ε_medium_* is the permittivity of the suspending medium, *E_rms_* is the root-mean-square of the applied electric field, and *f_CM_* is the Clausius–Mossotti factor of the cells, describing their polarization with respect to the surrounding medium.

Comprehensive numerical simulations were performed using ANSYS Fluent 6.3 software package (ANSYS Inc., Canonsburg, USA) to characterize the performance of DEP system, as detailed in Text S1 and Figure S1. Only three pairs of microelectrodes are incorporated in the numerical model to minimize the computational time. Simulations were conducted by applying a sine signal of 24 V_p-p_ and 5 MHz to microelectrodes and using a medium conductivity of 0.03 S/m.

Our results indicated that electric field increases smoothly along the microelectrodes, reaching a peak of 5.13×10^5 ^V/m at the tips (Fig. 1B). This exerted a maximum DEP force of 3.93 nN to the viable cells moving along the tips. The production of such strong electric fields heated the surrounding medium due to Joule heating effect [46], leading to a maximum temperature of 33°C at the tip region (Fig. 1C). This changed the local permittivity of the medium and induces a dielectric force [46], which dragged the medium towards the high temperature regions of the field (the tip region). This led to formation of two counter-rotating electro-thermal vortices within the PDMS chamber (Fig. 1C). A maximum velocity of 54 µm/s was calculated at the tip region. The vortices acted as conveyor belts and pushed the suspending cells towards the microelectrodes where they could be immobilized under the DEP force [43].

### Preparation of Non-budding Viable Yeast Cells

*Saccharomyces cerevisiae* yeast cells (powder, Sigma-Aldrich) were chosen as model cells. For non-budding viable cells sample preparation, a 1 M sorbitol solution was prepared and its medium conductivity was adjusted to 0.03 S/m by adding ∼40 µL of phosphate buffered saline (PBS). This slightly reduced the osmolarity of the sorbitol from 1214 mOSM to 1208 mOSM, as measured using an osmometer (Osmomat 030, Genotec). Next, 4 mg of cell powder was mixed with 8 mL of sorbitol/PBS buffer. The cell suspension was further subjected to ultrasonic water bath at 37°C for 30 minutes to prevent the agglomeration of cells. The optical density (OD600) of the cell suspension was measured and sorbitol/PBS buffer was added to adjust the OD600 value to 1.0 (∼3×10^7^ cell/mL).

### Preparation of Budding Yeast Cells

1 mg *Saccharomyces cerevisiae* yeast cells powder was cultured in 5 mL YPD (1% yeast extract, 2% peptone, 2% glucose, osmolarity value is 270 mOSM) at room temperature for 8 h. The growth of cells was monitored using inverted microscope every 2 h. The optical density (OD600) of the budding yeast suspension was measured and YPD buffer was added to adjust the OD600 value to 1.0. The cells were later washed with DI water and re-suspended in the sorbitol/PBS buffer.

### Preparation of Cell Fixation Medium

400 mg paraformaldehyde (PFA) powder (Sigma-Aldrich) was added into 10 mL DI water. The suspension was heated while stirring at 60°C. One droplet of 1 M NaOH solution was added to clear the suspension. Next, 500 mg glucose powder was added into the solution and the pH of the suspension was later adjusted to 7.2 to obtain a low conductivity 4% PFA cell fixation medium.

### Preparation of Lyticase Buffer

10 mg Lyticase powder (Sigma Aldrich, 200 U/mg) was firstly suspended into 200 µL deionised water. Next, the Lyticase buffer was prepared by adding 1 µL of the suspended Lyticase into 5 mL sorbitol/tris buffer (1 M sorbitol, 1 mM EDTA, 10 mM Tris buffer, pH 7.5), the final concentration of Lyticase in the buffer was 2 U/mL and the medium conductivity measured was around 0.035 S/m.

### Protocol for preparing yeast cells for SEM imaging

The following procedure was followed to prepare the cells for SEM imaging. Take the immobilization of non-budding cells as an example. The sample preparation procedures were divided into three major stages, which are immobilization, chemical treatments/fixation and dehydration. The detailed operating protocol for each stage is given below:

#### Stage 1: Immobilization

**Figure 2:**
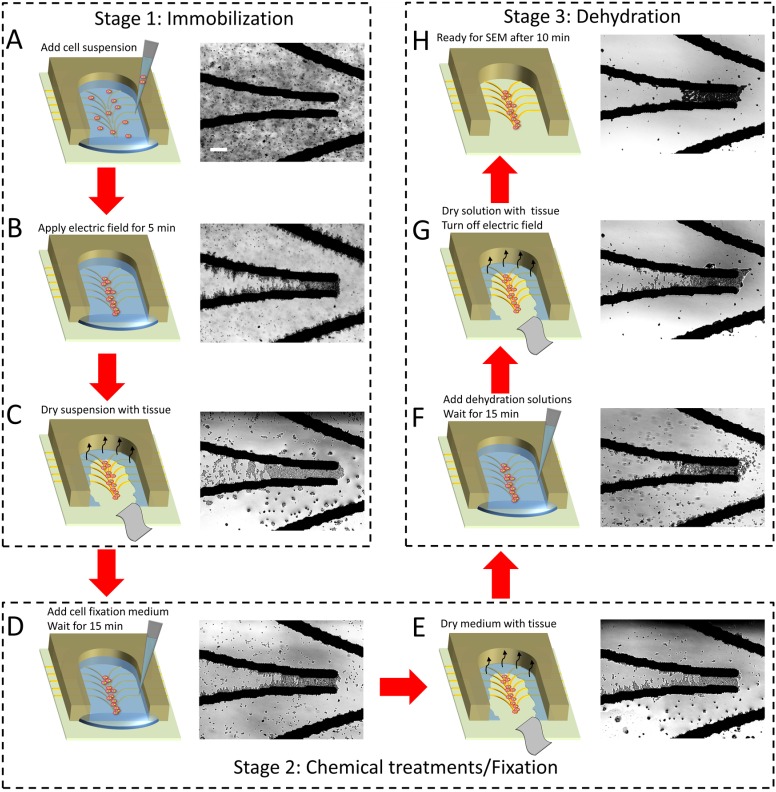
Protocols for obtaining immobilized cells for SEM. (A) Add cell suspension into the PDMS chamber. (B) Apply electric field and immobilize cells between the microelectrodes. Immobilized cell density can be adjusted by varying the electric field application period. (C) Dry the suspension with a lint-free cotton wipe. (D) Add media containing chemicals into the PDMS chamber for cell treatment. (E) Dry the medium with a lint-free cotton wipe. (F) Add dehydration solutions to the PDMS chamber. (G) Dry the dehydration solutions with a lint-free cotton wipe. Turn off the electric field and leave the sample for 10 minutes at room temperature to let the remained medium evaporate. (H) The sample is ready for SEM when all liquid is evaporated. Scale bar is 100 µm.

First, 30 µL of the non-budding yeast cell suspension (with 0.03 S/m medium conductivity) was added into the PDMS chamber. Yeast cells distributed evenly in the notch before the activation of electric field (Fig. 2A). Next, a sinusoidal signal with the magnitude and frequency of 24 V_p-p_ and 5 MHz, respectively, was applied to energize the microelectrodes. Under these conditions, the viable yeast cells experienced a strong positive DEP response and were immobilized between the microelectrodes (Fig. 2B). Alternatively, any possible non-viable cells contained in the suspension experienced a negative DEP force and were repelled from the microelectrodes. The electro-thermal vortices played a crucial role in driving the suspended cells towards the microelectrodes (Fig. 1C).

In our work, the desired density of immobilized cells was achieved in 5 minutes. This period can be varied to change the density of cells (see Figure S2). Next, a 3×3 cm lint-free cotton wipe (LymTech) was applied to the entrance of the notch to absorb the suspending medium. The capillary action of the lint-free wipe allows us to continuously and efficiently remove the suspending medium, leaving only a thin layer of medium along the side walls of the notch as shown in Fig. 2C. Some dislodgement of immobilized cells may occur during the drying step while the undesired non-viable cells can be washed away by the capillary force generated by the wipe.

#### Stage 2: Chemical Treatments/Fixation

30 µL of the desired chemicals was later added into the PDMS chamber to treat the cells for 15 min (Fig. 2D). The dislodged cells were re-immobilized between the electrodes during this process. Similarly, the medium was removed by applying a lint-free cotton wipe to the entrance of the notch (Fig. 2E). Applying the same procedures shown in Fig. 2D to 2E, the cell fixation medium was applied to the PDMS chamber.

#### Stage 3: Dehydration

**Figure 3:**
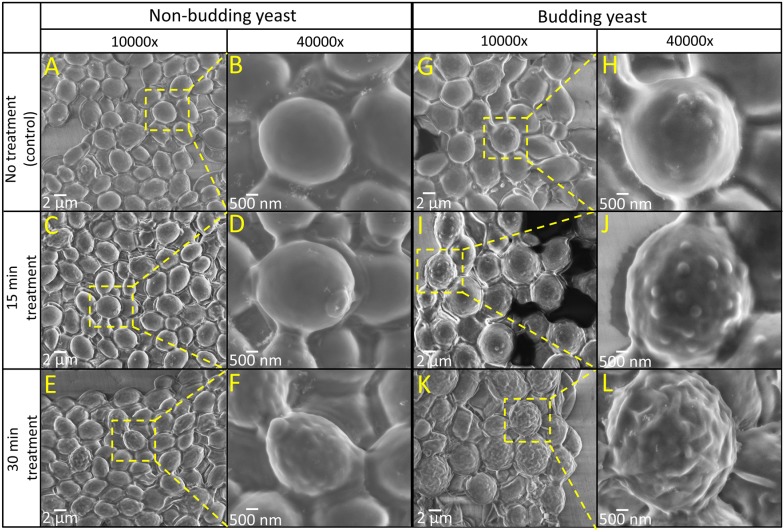
SEM images for both non-budding and budding yeast cells before and after Lyticase treatment at the magnifications of 10000× and 40000×.

30%, 50% and 70% ethanol solutions were added to the PDMS chamber sequentially (each for 5 min) to gradually dehydrate and fix the immobilized cells (Fig. 2F). The frequency of the applied sinusoidal signal was reduced to 100 kHz to allow the cells experience positive DEP response in the ethanol solution. Finally, after removing the solution using a lint-free cotton wipe (Fig. 2G), the electric field was turned off and the DEP platform was left at the room temperature for 10 minutes to ensure the full evaporation of the medium left inside the notch (Fig. 3H). The entire sample preparation process took around 45 minutes, which was two times shorter than that required in our previous protocol [43]. After finishing above procedure, the immobilized cells were subjected to SEM imaging.

### Experimental Setup and SEM Imaging

The conductivity of the suspensions was measured using a high precision conductivity meter (ECTestr11+, Eutech Instruments). The response of cells was observed with an inverted optical microscope (Nikon Eclipse, TE 2000). Sinusoidal wave signal was generated by a signal generator (Tabor, 2572A 100 MHz Dual-Channel) to energize the microelectrodes with one of the electrodes grounded. The DEP platform was placed on a computer controlled specimen stage to continuously monitor the treatment process (see Figure S3).

Following cell immobilization and buffer aspiration, high magnification and resolution images were taken using a scanning electron microscope (FEI Nova NanoSEM). A Helix gaseous secondary electron detector was implemented to achieve the SEM imaging under low vacuum mode. Resolution of the SEM has been adjusted at 3.0 spot size using 5 kV acceleration in 0.6 Torr (∼80 Pa) vacuum environment, enabling charge-free imaging and analysis of fully hydrated specimens.

## Results and Discussion

The developed system was utilized to study the morphological changes of non-budding and budding yeast cells following treatment with Lyticase, which is a complex of endoglucanase and protease that catalyzes removal of yeast cell wall [47].

Fig. 3A–B show the SEM images of immobilized non-budding viable yeast cells at 10000×and 40000× magnifications, respectively, obtained following the immobilization/fixation/dehydration procedures depicted in Fig. 2. Rather than single cells, clusters of cells were immobilized close to each other and located adjacent to the microelectrodes. Compared to conventional SEM, implementation of the DEP platform together with the high-resolution SEM under low vacuum mode, allowed us to observe the cell surface at a very high magnification with minimum excessive damage of cells and without the need to coat the cells with conducting materials. For comparison, the SEM images of immobilized yeast cells without using dielectrophoresis are also given in Figure S4. No significant difference was observed between the ultrastructure of cells immobilized without/with dielectrophoresis, indicating that the presence of electric filed in such a short period of time did not affect the morphology of yeast cells.

Fig. 3C–F show the SEM images of the immobilized non-budding yeast cells treated with Lyticase for 15 and 30 min, respectively. The images were obtained following the immobilization/stimulation/fixation/dehydration procedures depicted in Fig. 2. The Lyticase buffer was applied to the cells before applying the cell fixation medium. The morphology of yeast cells should change significantly when converting them to protoplasts following the removal of the cell wall, exhibiting smooth cell surface with characteristic invaginations [48]. However, our results indicate no significant changes on the surface of Lyticase treated non-budding yeast cells after 15 min. After 30 min treatment, a very small portion of cells (<10%) exhibited invaginations on the surface, indicating the low protoplast conversion efficiency for the non-budding yeast cells, which is in line with other reports [47]–[49]. This can be attributed to the low cell wall porosity of stationary yeast cells, making the cell less susceptible to Lyticase digestion [49].

Alternatively, Fig. 3G–H show the SEM images of budding yeast cells following the immobilization/fixation/dehydration procedures shown in Fig. 2. Budding yeast cells were obtained by culturing the non-budding yeast cells in YPD solution for 8 h, as described in the Materials and Methods section. Compared to non-budding cells, the size of budding cells was ∼1.5–2.0 times larger. More importantly, unlike non-budding yeast, the morphology of budding yeast changed significantly following the 15 min treatment with the Lyticase, with the appearance of blebs on the surface (Fig. 3I–J). The formation of blebs was a characteristic feature of cell injury and is reported as a protective mechanism to trap the damaged segments of the cell plasma membrane [50], [51]. After 30 min treatment, more than 89±7% of cells exhibited invaginations on the surface, indicating the high protoplast conversion efficiency for the budding yeast cells, as shown in Fig. 3K–L. The high protoplast conversion efficiency obtained for budding yeast cells can be attributed to the fact that the cell wall porosity was maximal in the early exponential phase, suggesting that the protoplast conversion induced by Lyticase was affected by cell growth conditions [49]. Cell lysis was observed for budding yeast cells after 40 min of Lyticase treatment, however, no significant change was observed for the non-budding yeast cells.

We further conducted off-chip experiments to examine the protoplast conversion efficiency by monitoring the cell lysis spectrophotometrically. Here, the optical density of the cell suspension at the wavelength of 600 nm was calculated as: OD600 = −log(I_out_/I_in_), where I_out_ and I_in_ are the intensity of the light after and before the cell suspension, respectively, while the protoplast conversion efficiency was calculated as (1−OD600)×100%. In doing so, we added 5% sodium dodecyl sulfate (SDS) solution to both non-budding and budding yeast cell suspensions treated with 2 U/mL Lyticase. The addition of SDS leads to the lysis of the cells whose cell wall has been removed by Lyticase, and reduces the optical density of the cell suspension.

**Figure 4:**
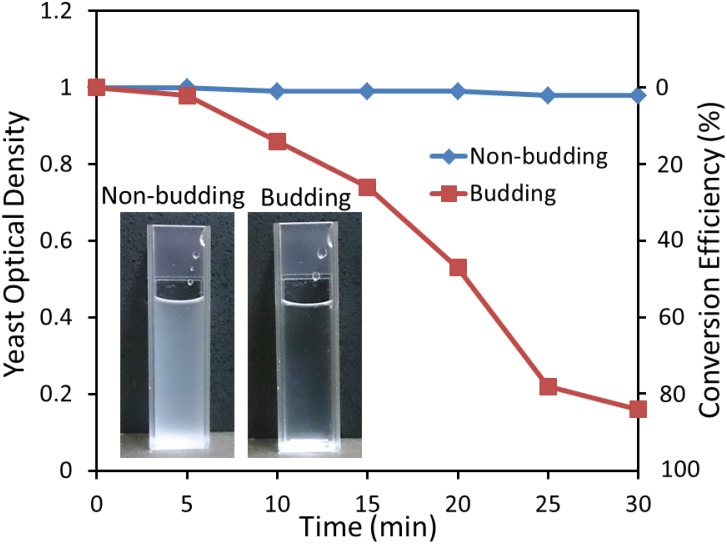
Optical density and protoplast conversion efficiency *vs* time plot for Lyticase treated non-budding and budding yeast cells after adding 5% SDS solution. The two insets show the non-budding and budding yeast suspensions following a 30 min treatment with 2 U/mL Lyticase and 5% SDS.

Fig. 4 shows the dynamic changes of the optical density and the protoplast conversion efficiency of the cell suspensions within 30 min. The protoplast conversion efficiency of non-budding and budding yeast cells following a 30 min Lyticase treatment was calculated as <1% and 84%, respectively, which is in line with the results obtained using SEM (Fig. 3 E and K).

The capability of the developed system was further demonstrated by studying the interaction of viable yeast cells with micro/nano materials including 850 nm polystyrene particles and multi-walled carbon nanotubes (MWCNTs), as shown in Text S2 and Figures S5–7. High resolution SEM images were obtained to clearly show how cells interact with such micro/nano materials.

## Conclusion

The developed protocol greatly reduces the complexity of conventional methods and enables high-resolution SEM imaging of cells. Most importantly, this protocol allows the on-chip stimulation and fixation of immobilized cells, as well as rapid and proper dehydration of the sample. These advantages were demonstrated with the experiments interfacing cells with SEM at low vacuum mode for comparing the morphological changes of non-budding and budding yeast cells following Lyticase treatment.

Based on obtained results, we strongly envisage prospective applications of the developed protocol for study of cell morphological changes using SEM when subjected to conditions including apoptosis [1], chemical stimulation [2]–[8], as well as physical stimulation such as change of environment temperature [12] or mechanical forces [9]. We also prospect further development of this protocol for instant characterization of interfacing nanomaterials with cells with huge opportunities for drug delivery and biosensor applications [2].

## Supporting Information

Figure S1
**Contours of (a)** ***E*** **and (b)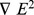 produced by the curved microelectrodes at 30 V_p-p_, obtained by numerical simulations.**
(TIF)

Figure S2 **Elongating the duration of experiment increases the density of trapped cells.** The immobilization of viable cells when conductivity of the medium are set to 0.03 S/m while the magnitude and frequency of the AC signal are set to 24 V_p-p_ and 5 MHz. Scale bar is 150 µm.
(TIF)

Figure S3 **The DEP system was placed on a specimen stage to continuously monitor the treatment process.** The DEP system consists of an open-top PDMS channel assembled onto a DEP platform. The wires were bonded to the microelectrode pads using aluminum tapes.
(TIF)

Figure S4 **(A) SEM images for non-budding yeast cells without using dielectrophoresis.** After applying the cell fixation medium (4% PFA), the cells were dehydrated with ethanol series on a carbon substrate and the SEM images were obtained under low vacuum mode. (B) SEM images for non-budding yeast cells using dielectrophoresis. No significant difference is observed between the ultrastructure of cells immobilized without/with dielectrophoresis.
(TIF)

Figure S5 **Schematic of the multi-layer structure of a yeast cell, consisting of cytoplasm, plasma membrane and an outer wall.**
(TIF)

Figure S6 **The Re[** ***f_CM_*** **] spectra of (A) viable yeast cells, (B) polystyrene particles, (C) MWCNTs and (D) MWCNTs coated viable yeast cells in a medium with the conductivity of 0.03 S/m.**
(TIF)

Figure S7 **SEM images for (A) viable yeast, (B) viable yeast mixed with 850 nm polystyrene particles, and (C) viable yeasts coated with MWCTNs.** (D) shows the white field and fluorescent images for viable yeast coated with Rhodamine 123 conjugated MWCNTs.
(TIF)

References S1 (DOCX)

Text S1 (DOCX)

Text S2 (DOCX)
