# Supplementary figures and images for: High Resolution Scanning Electron Microscopy of Cells Using Dielectrophoresis
Source: PLoS One. 2014 Aug 4;9(8):e104109. doi: 10.1371/journal.pone.0104109 (PMC4121316; doi:10.1371/journal.pone.0104109)

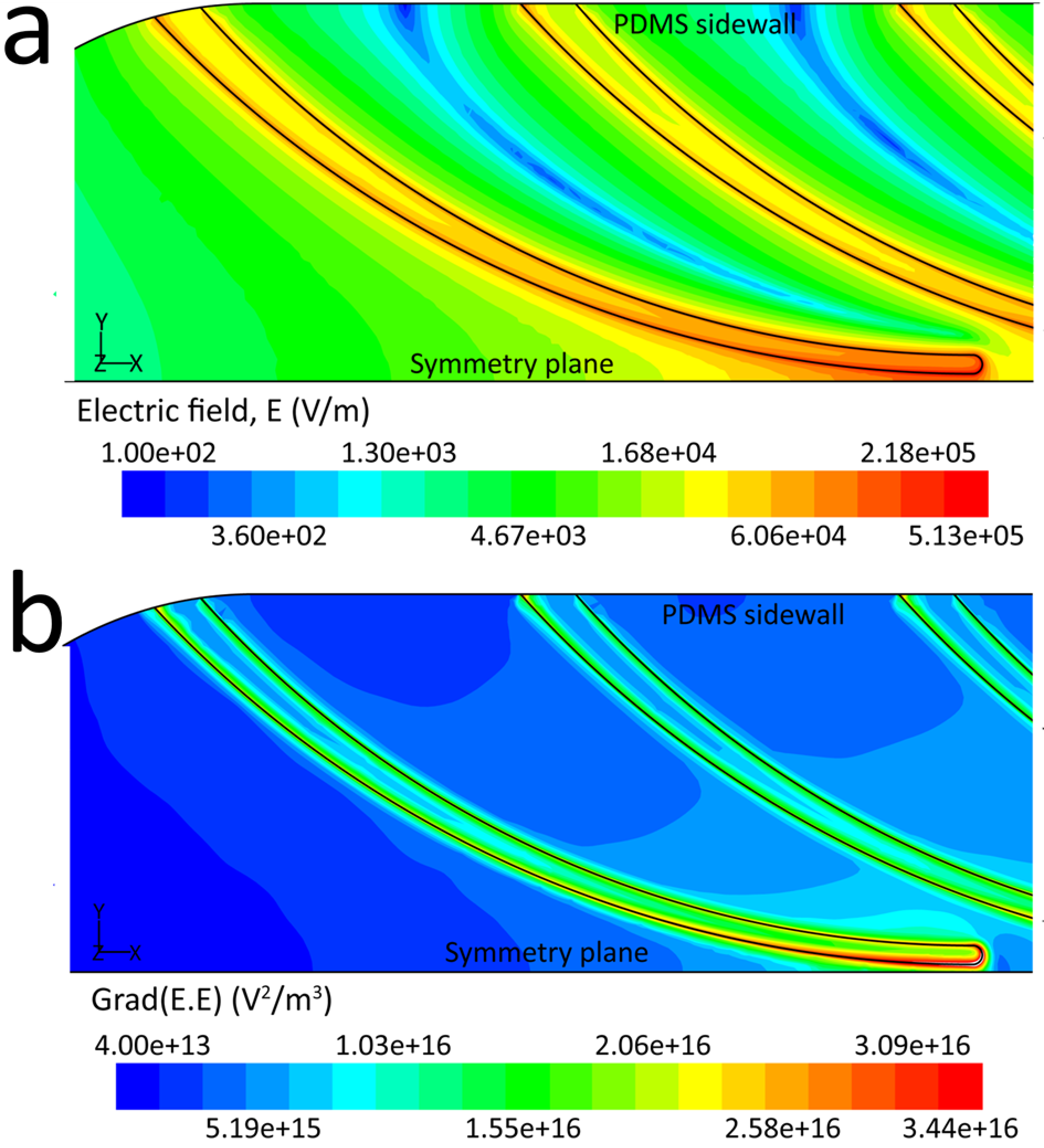

Supplement: Figure S1 — Contours of (a) E and (b) produced by the curved microelectrodes at 30 Vp-p, obtained by numerical simulations. (TIF) [file pone.0104109.s001.tif]

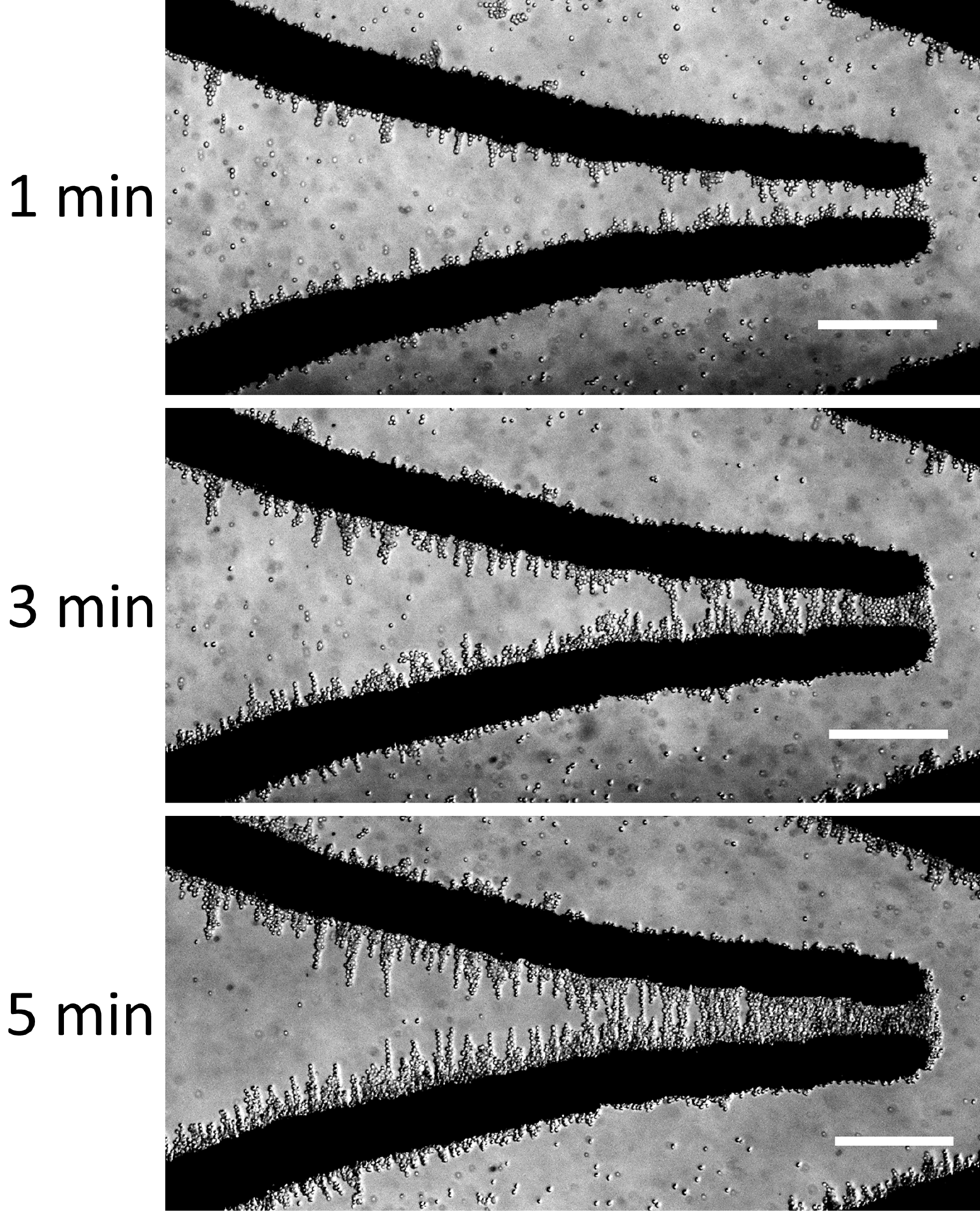

Supplement: Figure S2 — Elongating the duration of experiment increases the density of trapped cells. The immobilization of viable cells when conductivity of the medium are set to 0.03 S/m while the magnitude and frequency of the AC signal are set to 24 Vp-p and 5 MHz. Scale bar is 150 µm. (TIF) [file pone.0104109.s002.tif]

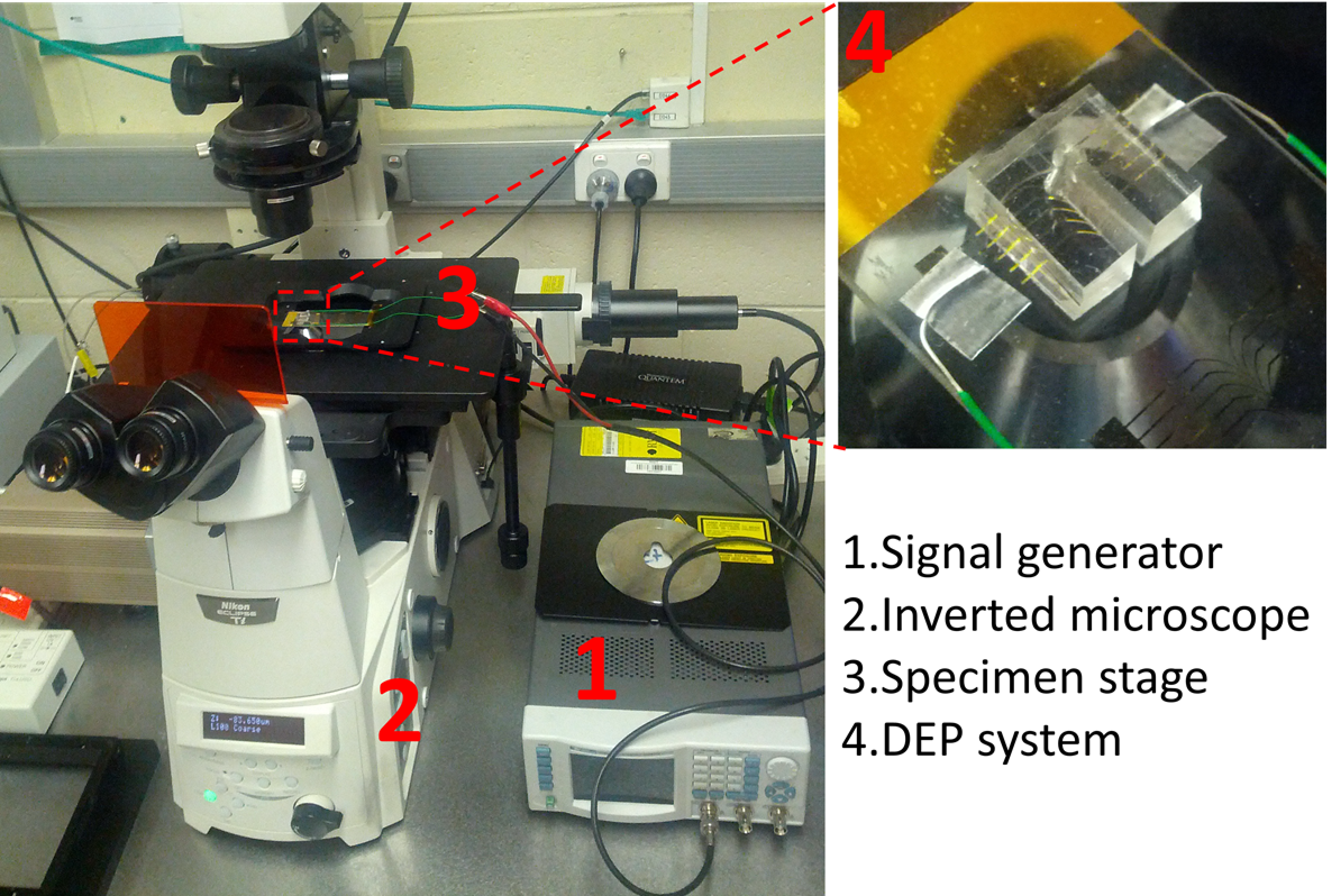

Supplement: Figure S3 — The DEP system was placed on a specimen stage to continuously monitor the treatment process. The DEP system consists of an open-top PDMS channel assembled onto a DEP platform. The wires were bonded to the microelectrode pads using aluminum tapes. (TIF) [file pone.0104109.s003.tif]

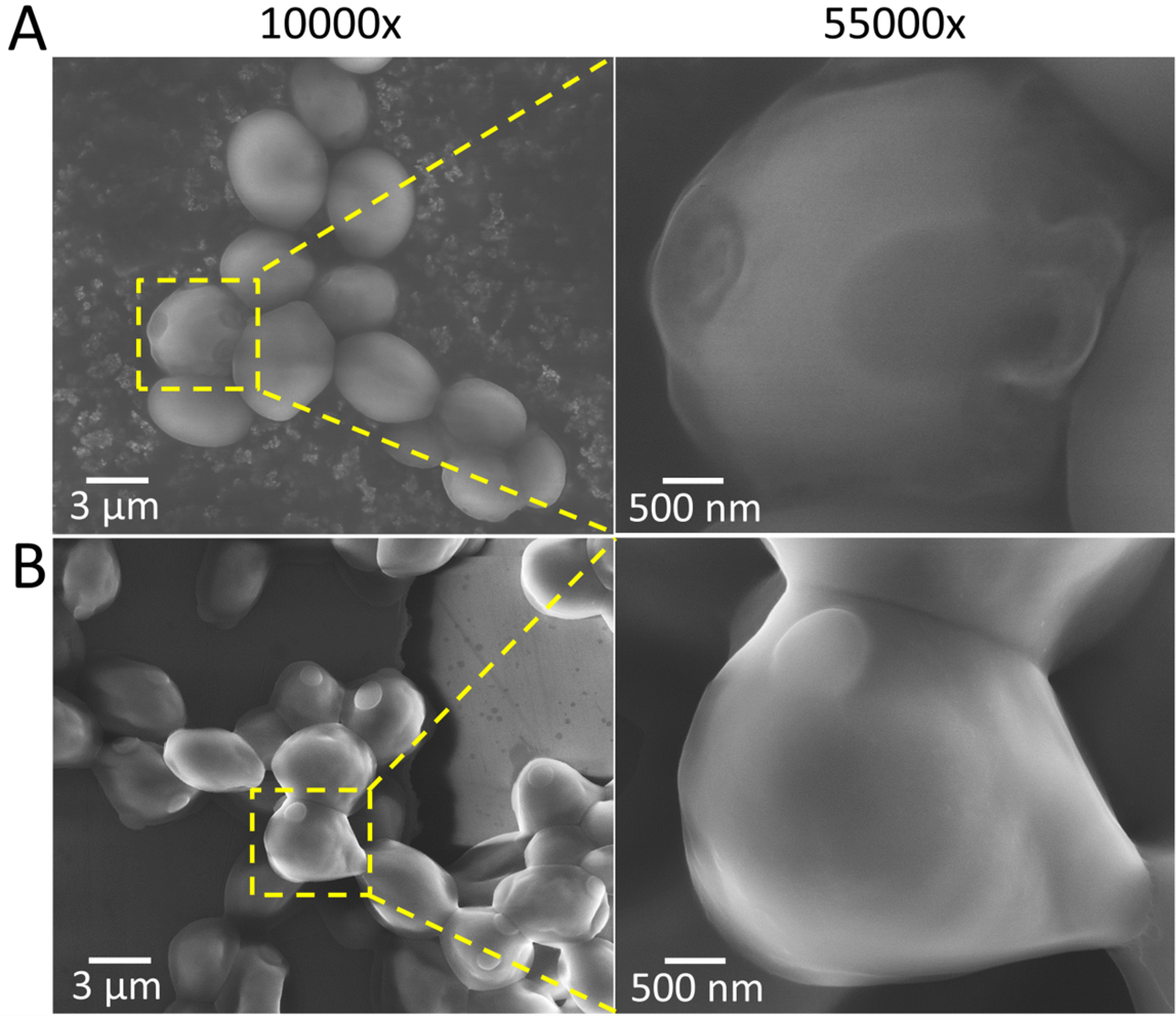

Supplement: Figure S4 — (A) SEM images for non-budding yeast cells without using dielectrophoresis. After applying the cell fixation medium (4% PFA), the cells were dehydrated with ethanol series on a carbon substrate and the SEM images were obtained under low vacuum mode. (B) SEM images for non-budding yeast cells using dielectrophoresis. No significant difference is observed between the ultrastructure of cells immobilized without/with dielectrophoresis. (TIF) [file pone.0104109.s004.tif]

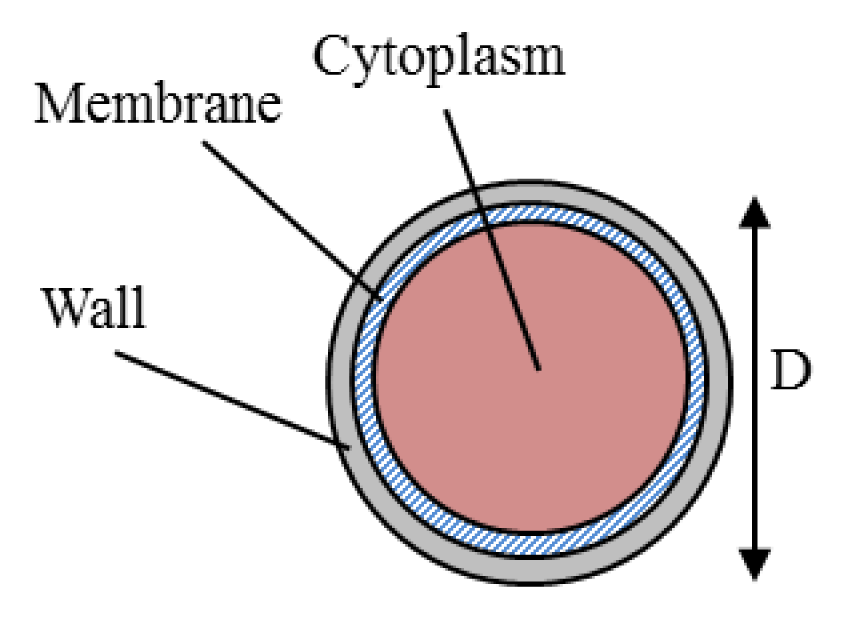

Supplement: Figure S5 — Schematic of the multi-layer structure of a yeast cell, consisting of cytoplasm, plasma membrane and an outer wall. (TIF) [file pone.0104109.s005.tif]

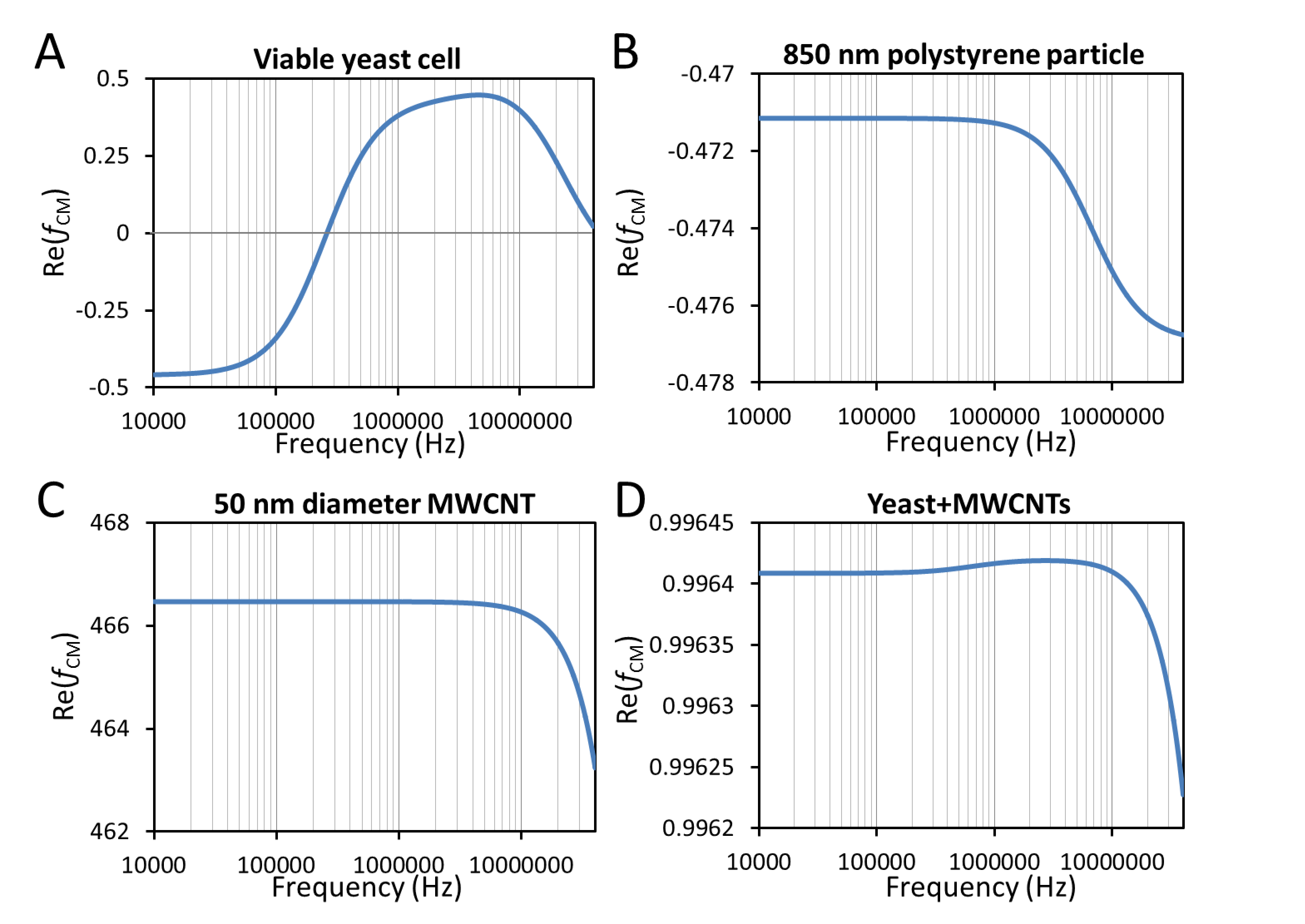

Supplement: Figure S6 — The Re[ fCM ] spectra of (A) viable yeast cells, (B) polystyrene particles, (C) MWCNTs and (D) MWCNTs coated viable yeast cells in a medium with the conductivity of 0.03 S/m. (TIF) [file pone.0104109.s006.tif]

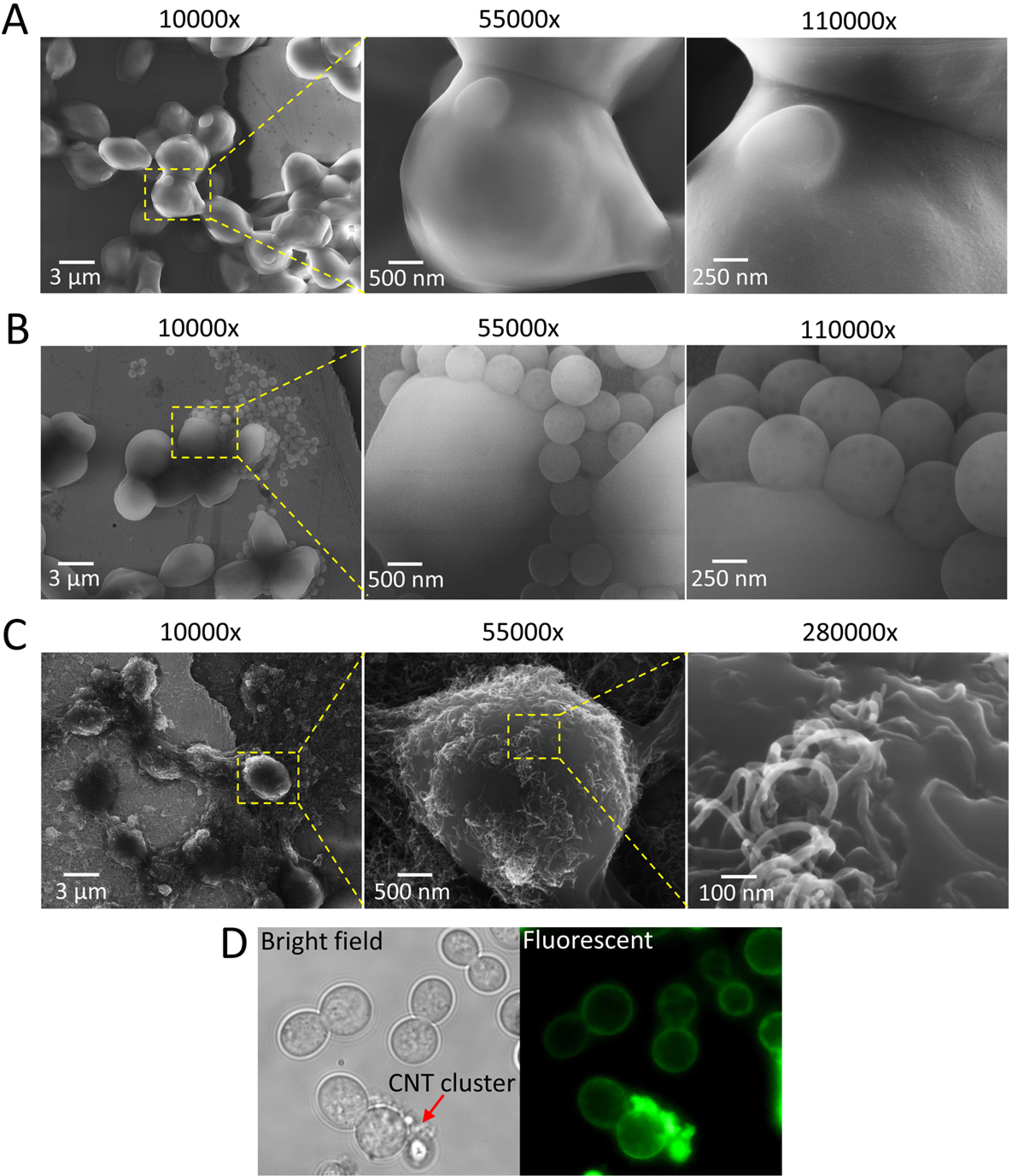

Supplement: Figure S7 — SEM images for (A) viable yeast, (B) viable yeast mixed with 850 nm polystyrene particles, and (C) viable yeasts coated with MWCTNs. (D) shows the white field and fluorescent images for viable yeast coated with Rhodamine 123 conjugated MWCNTs. (TIF) [file pone.0104109.s007.tif]
