# Supplementary material for: High Resolution Scanning Electron Microscopy of Cells Using Dielectrophoresis
Source: PLoS One. 2014 Aug 4;9(8):e104109. doi: 10.1371/journal.pone.0104109 (PMC4121316; doi:10.1371/journal.pone.0104109)
Supplement: References S1 — (DOCX) [file pone.0104109.s008.docx]

**Supporting Information Reference**

[1] Morgan H.; Green N. G. *AC Electrokinetics: Colloids and Nanoparticles*., Research Studies Press: Philadelphia, PA, 2003.

[2] Urdaneta, Smela, M. E., *Electrophoresis* 2007, *28*, 3145-3155.

[3] Cui, L., Holmes, D., Morgan, H., *Electrophoresis* 2001, *22*, 3893–3901.

[4] Krupke, R., Hennrich, F., Kappes, M. M., Lohneysen, H. V., *Nano Lett.* 2004, *4*, 1395–1399.

[5] Wang L., Zhang L., Xue X., Ge G., Liang X., *Nanoscale* 2012, *4*: 3983-3989.
