# Supplementary material for: High Resolution Scanning Electron Microscopy of Cells Using Dielectrophoresis
Source: PLoS One. 2014 Aug 4;9(8):e104109. doi: 10.1371/journal.pone.0104109 (PMC4121316; doi:10.1371/journal.pone.0104109)
Supplement: Text S1 — (DOCX) [file pone.0104109.s009.docx]

**Text S1. Numerical simulation of electric field**

In order to calculate the contours of electric field, the Laplace equation was solved within the PDMS chamber by applying appropriate electric potentials at the microelectrodes while zero electric flux at other surfaces of the chamber, including the bottom, top and sidewalls:

(S1)

Next, the electric field was calculated by differentiating the electric potential:

(S2)

Finally, the DEP forces were obtained by calculating the gradient of electric field square:

$F_{DEP}\propto\nabla E^{2}$ (S3)

Figure S1 illustrates the contours of *E* and $\nabla E^{2}$produced by the curved microelectrodes at
30 V_p-p_, obtained by numerical simulations.

Next, we solved the differential equations governing the balance of mass, momentum and energy to obtain the variations of velocity and temperature for the buffer within the PDMS chamber:

| $\nabla\cdot\vec{\text{U}} = 0$ | (S4) |
| --- | --- |
| $\begin{matrix} \rho_{medium} \vec{U}\cdot\nabla\vec{U}= -\nabla P + \nabla.(\mu_{medium} \nabla\vec{U}) \\ -0.5 \left(\frac{\partial\sigma}{\sigma\partial T} -\frac{\partial\varepsilon}{\varepsilon\partial T}\right) \frac{\varepsilon}{1+(\omega\tau)^{2}}(\nabla T\cdot E_{rms}) E_{rms} + 0.25 \frac{\partial\varepsilon}{\partial T} E_{rms}^{2} \nabla T \end{matrix}$ | (S5) |
| $\rho_{medium} c_{medium} \vec{U}\cdot\nabla T= \nabla.(k_{medium} \nabla T)+ \sigma_{medium} E_{rms}^{2}$ | (S6) |

where $\vec{U}$is the velocity vector, *P* is the local pressure, and *T* is the local temperature of the buffer, while *ρ_medium_, µ_medium_, c_medium_*, and *k_medium_* are the density, dynamic viscosity, specific heat and thermal conductivity of the buffer, respectively. On the other hand, *σ* and *ε* are conductivity and permittivity of the buffer, $\tau=\varepsilon/\sigma$ is the charge relaxation time of the medium, and the variations of conductivity and permittivity of the buffer can be expressed as below [1]:

*(1/σ)(∂σ/∂T)*= +0.02 ˚C^-1^ (S7)

*(1/ε)(∂ε/∂T)*= −0.004 ˚C^-1^ (S8)

The energy equation includes a source term to represent the Joule heating effect. This increases the temperature at the vicinity of microelectrodes. The momentum equation includes two source terms to represent the electro-thermal forces within the chamber, which lead to formation of electro-thermal vortices. The source terms include the Coulomb force (the term at the left) and the dielectric force term (the term at the right). The Coulomb force dominates at low frequencies while the dielectric force dominates at high frequencies [1]. The results of numerical simulation are shown in Figure 1C.
