# Supplementary material for: High Resolution Scanning Electron Microscopy of Cells Using Dielectrophoresis
Source: PLoS One. 2014 Aug 4;9(8):e104109. doi: 10.1371/journal.pone.0104109 (PMC4121316; doi:10.1371/journal.pone.0104109)
Supplement: Text S2 — (DOCX) [file pone.0104109.s010.docx]

**Text S2. SEM for yeast cells interact with micro/nano materials**

The capability of the developed system was further demonstrated by studying the interaction of viable yeast cells with micro/nano materials including 850 nm polystyrene particles and multi-walled carbon nanotubes (MWCNTs). The DEP response of yeast cell, polystyrene particle and MWCNTs were studied. Yeast cell can be considered as a homogenous spherical structure, the Clausius-Mosotti factor *f_CM_* can be calculated as below [1]:

| $f_{CM}=\frac{\varepsilon_{cell}^{*}- \varepsilon_{medium}^{*}}{\varepsilon_{cell}^{*} + 2 \varepsilon_{medium}^{*}}$ | (S9) |
| --- | --- |
| $\varepsilon^{*} = \varepsilon-\frac{i\sigma}{\omega}, i=\sqrt{-1}$ | (S10) |

where *ε** is the complex permittivity, *ε* is the permittivity, *σ* is the electrical conductivity, and *ω* is the angular frequency of the applied AC signal. Yeast cells have a multi-layer structure consisted of cytoplasm, plasma membrane and an outer wall, as shown in Figure S5 [2].

The common approach to predict the *f_CM_* of yeast cells is to apply the double-shell spherical model. In doing so, first the equivalent complex permittivity of the cytoplasm and the surrounding membrane is calculated from Eq. S11. Next, the equivalent complex permittivity of the cytoplasm-membrane and the surrounding wall is calculated from Eq. S12 to be substituted in Eq. S9.

| $\varepsilon_{cytoplasm-membrane}^{*}= \varepsilon_{membrane}^{*} \frac{\left[\frac{r-t_{wall}}{r-t_{wall}-t_{membrane}}\right]^{3}+2 \left[\frac{\varepsilon_{cytoplasm}^{*}- \varepsilon_{membrane}^{*}}{\varepsilon_{cytoplasm}^{*}+ 2 \varepsilon_{membrane}^{*}}\right]}{\left[\frac{r-t_{wall}}{r-t_{wall}-t_{membrane}}\right]^{3}-\left[\frac{\varepsilon_{cytoplasm}^{*}- \varepsilon_{membrane}^{*}}{\varepsilon_{cytoplasm}^{*}+ 2 \varepsilon_{membrane}^{*}}\right]}$ | (S11) |
| --- | --- |
| $\varepsilon_{cell}^{*}= \varepsilon_{wall}^{*} \frac{\left[\frac{r}{r-t_{wall}}\right]^{3}+2 \left[\frac{\varepsilon_{cytoplasm-membrane}^{*}- \varepsilon_{wall}^{*}}{\varepsilon_{cytoplasm-membrane}^{*}+ 2 \varepsilon_{wall}^{*}}\right]}{\left[\frac{r}{r-t_{wall}}\right]^{3}-\left[\frac{\varepsilon_{cytoplasm-membrane}^{*}- \varepsilon_{wall}^{*}}{\varepsilon_{cytoplasm-membrane}^{*}+ 2 \varepsilon_{wall}^{*}}\right]}$ | (S12) |

The geometric and dielectric properties of viable yeast cells are given in the following table, and Figure S6A shows the Re[*f_CM_*] spectra of viable yeast cells obtained by Eq. (S9–S12) within a medium with the conductivity of 0.03 S/m. The cells experience the maximum DEP force when the frequency is ~5 MHz.

Geometric and dielectric properties of viable yeast cells

| **Properties** | **Viable Cells** |
| --- | --- |
| *r* (µm) | 4 |
| *σ_cytoplasm_* (S/m) | 0.2 |
| *ε_cytoplasm_* (F/m) | 50 ε_0_ |
| *σ_membrane_* (S/m) | 25×10^-8^ |
| *ε_membrane_* (F/m) | 6 ε_0_ |
| *t_membrane_* (nm) | 8 |
| *σ_wall_* (S/m) | 14×10^-3^ |
| *ε_wall_* (F/m) | 60 ε_0_ |
| *t_wall_* (nm) | 220 |

For polystyrene particle, *f_CM_* can be calculated using Eq. S9 by replacing *ε^*^_cell_* to *ε^*^_particle_*. Assuming the polystyrene microparticles have an overall conductivity of 3.5×10^-4^ S/m (composed of core and induced surface conductance) [3] and a relative permittivity of 2.5 [3], Figure S6B shows the Re[*f_CM_*] spectra calculated for the particles within a medium with the conductivity of 0.03 S/m. The polystyrene particles experience strong negative DEP response across the frequency range.

For calculating the Re[*f_CM_*] of MWCNTs, the shape of the MWCNTs can be assume to be cylindrical and the Re[*f_CM_*] is calculates as below [4]:

| $f_{CM}=\frac{\varepsilon_{MWCNT}^{*}- \varepsilon_{medium}^{*}}{\varepsilon_{medium}^{*} + (\varepsilon_{MWCNT}^{*}- \varepsilon_{medium}^{*})L}$ | (S13) |
| --- | --- |

where *L* is the depolarisation factor of the MWCNTs deﬁned as (2*r_CNT_*/*l*)^2^[in(*l*/*r*)-1][5], *r_CNT_* is the radius of the MWCNTs and *l* is the length of the MWCNTs. The MWCNTs have a diameter of ~25 nm and a length of ~2 μm. Assuming the conductivity of the MWCNTs is 1000 S [4] and their relative permittivity is 500 [4], Re[*f_CM_*] spectra is obtained when the medium conductivity is 0.03 S/m, as shown in Fig. S6C. The MWCNTs experience strong positive DEP response across the frequency range.

For the case of MWCNTs coated cells, we assume the surface of the cells is coated with an extra layer of MWCNT coating. In this case, the equivalent complex permittivity of the MWCNTs coated cell is calculated from Eq. S14.

| $\varepsilon_{cell-MWCNT}^{*}= \varepsilon_{MWCNT}^{*} \frac{\left[\frac{r+2r_{MWCNT}}{r}\right]^{3}+2 \left[\frac{\varepsilon_{cell}^{*}- \varepsilon_{MWCNT}^{*}}{\varepsilon_{cell}^{*}+ 2 \varepsilon_{MWCNT}^{*}}\right]}{\left[\frac{r+2r_{MWCNT}}{r}\right]^{3}-\left[\frac{\varepsilon_{cell}^{*}- \varepsilon_{MWCNT}^{*}}{\varepsilon_{cell}^{*}+ 2 \varepsilon_{MWCNT}^{*}}\right]}$ | (S14) |
| --- | --- |

Replacing Eq. S14 into Eq. 9, the Re[*f_CM_*] spectra for the MWCNTs coated cells is calculated, as shown in Fig. S6D. This MWCNTs coating significantly change the DEP response of the cells and they experience strong positive DEP response across the frequency range.

Following the protocol shown in Fig. 2 in the main manuscript, we firstly obtain SEM images of immobilized non-budding viable yeast cells at 10000×, 55000× and 110000× magnifications, as shown in Fig. S7A.

We further used our DEP system to study the interface between the cells and the 850 nm polystyrene particles under SEM, as shown in Fig. S7B. Although the particles exhibited negative DEP response within a medium of 0.03 S/m conductivity, the amount of DEP force was much weaker compared to the drag force caused by electro-thermal vortices. Thus some of the moving particles could be trapped between the gaps of immobilized cells, as observed in our experiments. The interface between the particles and yeast cell surface can be clearly observed under SEM and our images reveal a small deformation over the cell surface to accommodate the settling particles (clearly shown at 110000× magnification in Fig. S7B).

We further demonstrate the capability of our DEP system for exploring the interaction between the cells and nanomaterials. In doing so, we coated yeast cells with multi-walled carbon nanotubes (MWCNTs) by adding 50 μL MWCNT suspension (0.5 mg/mL) into 450 μL yeast cell suspension.

Figure S7C show the SEM images of cells obtained following 5 min incubation with MWCNT and following the protocol given in Fig. 2. Cells were coated by a thin layer of MWCNTs. The drying procedure (Fig. 2) removed excess suspension in the notch, which prevented the uncoated MWCNTs dispersed in the solution to deposit onto the cell surface during the evaporation process. The attachment of the MWCNTs to the cell wall was further investigated by fluorescent microscopy. In doing so, fluorescent MWCNTs were prepared by mixing 500 μL of MWCNT suspension (0.5 mg/mL) with 40 μL of Rhodamine 123 (100 nM) for 2 h [5]. NAP-10 column (GE Healthcare) was used to remove the excess Rhodamine 123 molecules. Next, 50 μL of the Rhodamine 123 labelled MWCNT suspension was added into 450 μL of yeast cell suspension. The cells were washed for three times with 1 M sorbitol solution and the fluorescent images were obtained using an inverted microscope, as shown in Fig. S7D. The results indicate the attachment of Rhodamine 123 conjugated MWCNT to the surface of yeast cells.
